# Value of digital mammography in predicting lymphovascular invasion of breast cancer

**DOI:** 10.1186/s12885-020-6712-z

**Published:** 2020-04-03

**Authors:** Zhuangsheng Liu, Ruqiong Li, Keming Liang, Junhao Chen, Xiangmeng Chen, Xiaoping Li, Ronggang Li, Xin Zhang, Lilei Yi, Wansheng Long

**Affiliations:** 1 Department of Radiology, Jiangmen Central Hospital, Affiliated Jiangmen Hospital of Sun Yat-Sen University, No. 23 Haibang Street, Jiangmen, 529000 Guangdong China; 2 Department of Gastrointestinal Surgery, Jiangmen Central Hospital, Affiliated Jiangmen Hospital of Sun Yat-Sen University, Jiangmen, Guangdong China; 3 Department of Pathology, Jiangmen Central Hospital, Affiliated Jiangmen Hospital of Sun Yat-Sen University, Jiangmen, Guangdong China; 4 Department of Clinical Experimental Center, Jiangmen Central Hospital, Affiliated Jiangmen Hospital of Sun Yat-Sen University, Jiangmen, Guangdong China; 5 Department of Radiology, Foshan Hospital of Traditional Chinese Medicine, Foshan, Guangdong China

**Keywords:** Lymphovascular invasion, Breast cancer, Digital mammography

## Abstract

**Background:**

Lymphovascular invasion (LVI) has never been revealed by preoperative scans. It is necessary to use digital mammography in predicting LVI in patients with breast cancer preoperatively.

**Methods:**

Overall 122 cases of invasive ductal carcinoma diagnosed between May 2017 and September 2018 were enrolled and assigned into the LVI positive group (*n* = 42) and the LVI negative group (*n* = 80). Independent t-test and χ2 test were performed.

**Results:**

Difference in Ki-67 between the two groups was statistically significant (*P* = 0.012). Differences in interstitial edema (*P* = 0.013) and skin thickening (*P* = 0.000) were statistically significant between the two groups. Multiple factor analysis showed that there were three independent risk factors for LVI: interstitial edema (odds ratio [OR] = 12.610; 95% confidence interval [CI]: 1.061–149.922; *P* = 0.045), blurring of subcutaneous fat (OR = 0.081; 95% CI: 0.012–0.645; *P* = 0.017) and skin thickening (OR = 9.041; 95% CI: 2.553–32.022; *P* = 0.001).

**Conclusions:**

Interstitial edema, blurring of subcutaneous fat, and skin thickening are independent risk factors for LVI. The specificity of LVI prediction is as high as 98.8% when the three are used together.

## Background

Breast cancer metastasizes through lymphatic and blood vessels, which makes the patients susceptible to distant metastasis, postoperative recurrence and even death. Lymphovascular invasion (LVI) has never been revealed by preoperative scans. Diagnostic histopathology is needed to reveal LVI of cancer cells. Preoperative identification of LVI can help to predict the prognosis of patients with breast cancer, especially those with axillary node-negative breast cancer, and to develop adjuvant treatment plans in clinical settings [1, 2].

Digital mammography is one of the important imaging tools for breast cancer screening and diagnosis. There have been many reports on prediction of axillary lymph node metastasis [3–5] and on digital mammography screening [6–9]. However, there has been no report on predicting LVI of breast cancer based on imaging patterns of digital mammography, except a little literature in which MRI findings were used for prediction [2, 10–12].

It is necessary to use digital mammography, a more readily available imaging tool, in preoperative prediction of LVI in patients with breast cancer, and to evaluate the predictive specificity of the imaging findings and certain biomarkers detected by immunohistochemistry so as to better foresee disease progression and develop targeted treatment plans.

## Methods

### Clinical data

This single-center retrospective study enrolled 122 cases of invasive ductal carcinoma diagnosed between May 2017 and September 2018. Since the data were obtained from a picture archiving and communication system, patients’ informed consent was not required for this study. The inclusion criteria were: (1) modified radical mastectomy or breast-conserving surgery + axillary lymph node dissection; (2) diagnosis of invasive breast cancer confirmed by routine histopathological and immunohistochemical examinations; (3) no previous history of breast tumors or primary tumors in other locations; and (4) LVI status confirmed by immunohistochemistry. Patients were excluded if they had obscured lesions revealed by digital mammograms, were breastfeeding, had underwent lumbar puncture or radiotherapy prior to diagnosis, and had incomplete clinical data.

Mammographic lesions were confirmed either by core needle biopsies or by surgical pathology. All the patients underwent digital mammography preoperatively. All of them were female and aged between 26 and 77 with a median age of 45. They were assigned into the LVI positive group (*n* = 42) and the LVI negative group (*n* = 80).

### Digital mammography

GE (Senographe DS) and IMS (GIOTTO IMAGE) systems were used. Four standard body positions were imaged under automatic exposure conditions, and they were the right craniocaudal (RCC) view, left craniocaudal (LCC) view, right mediolateral oblique (RMLO) view and left mediolateral oblique (LMLO) view. When lesions were found in the axillary tail of Spence or near the cleavage, amplified imaging in these locations was done to reveal the lesions fully. The flat-panel detector of internal and external obliques was parallel to the pectoralis major muscle. According to the patients’ body shape, the projecting angle ranged from 40° to 65° and was usually 60°. The glands were fully unfolded, and the skin folds below the breasts and upper abdomen were within the mammographic field.

### Image analysis

The imaging characteristics of breast cancer lesions were described according to the 5th edition of ACR BI-RADS® Atlas published in 2013 [13]. The lesions were classified into four types: mass, calcification, architectural distortion, and asymmetry. There could be one or more masses. The masses could be round, lobulated, and irregular. Clear and sharp edges meant well circumscribed margins, clear and sharp edges obscured by glands meant obscured margins, while obscured and spiculated edges meant indistinct margins. Amorphous and fine polymorphic calcifications were included, while typically benign calcifications were excluded. Architectural distortion referred to abnormal deformation of breasts without any mass clearly revealed by imaging. History of trauma and surgery must be ruled out under this circumstance. Focal asymmetry was seen in two images, but lacks the outward border or a mass. There were only few cases of architectural distortion and focal asymmetry (both *n* < 5). Those cases were excluded to avoid inaccurate statistical results. And the lesion type was classified as mass and calcification only.

Associated features included nipple discharge, interstitial edema, blurring of subcutaneous fat layer, skin thickening, and axillary adenopathy (lymph nodes measuring > 1 cm in the long axis diameter with absence of hilus. If enlarged lymph nodes are new, they need to be clinically combined and further examined). Due to its absence in the LVI negative group (*n* = 0), nipple discharge was not included as a variable. Thickening of the skin means that the affected breast has localized or diffuse thickening of the skin greater than 2 mm in thickness. Interstitial edema and subcutaneous fat layer blurring are caused by the filling and dilation of lymphatic vessels and blood vessels in the breast, while the performance of the whole breast including subcutaneous fat layer is blurring, and multiple cord shadows are seen.

### Pathology

Surgically resected breast cancer specimens were fixed in 10% formaldehyde for 24 h, then were dehydrated and embedded in paraffin wax. Sections were prepared. Standard HE stain and streptavidin-peroxidase-biotin (SP) immunohistochemical method were performed. DAB detection systems were used. A score of 3 and more indicated Her-2 overexpression. Positive FISH result of Her-2 gene amplification also indicated overexpression when the score was 2 and more. Ki67 proliferative index (PI) of <10% indicated low expression level, while that of > 30% indicated high expression level. Ki67 PI of 10 to 30% indicated intermediate expression level [14].

The gold standard of this study was that LVI was defined as the intravenous tumor emboli and lymphatic tumor emboli detected by immunohistochemistry. These two kinds of tumor emboli were clinically referred to as intralymphovascular tumor emboli due to the difficulty of distinguishing them with pathological sections. The specimens were read by a senior pathologist who had been working on breast cancer for 21 years.

### Statistical analysis

The data were analyzed using SPSS Version 19.0. The quantitative data were expressed as means ± SDs. The independent t-test was done for intergroup comparisons. The count data were expressed as frequencies or rates, and the χ2 test or Fisher’s method was performed. *P* < 0.05 indicated statistically significant difference. The count data whose values were 0 were excluded from statistical analysis and listed only in table(s).

## Results

### General data and biomarkers

Table 1 presents the data about childbearing history, miscarriage history, history of other breast diseases, nipple discharge, CA153, age, ER, PR, HER-2, E-CAD, P53, and Ki-67. Difference in Ki-67 between the LVI positive group and the LVI negative group was statistically significant (*P* = 0.012), while no statistically significant differences were observed in the other factors mentioned.

**Table 1:** Comparison of patient characteristics according to lymphovascular invasion

Characteristics	Total	LVI = 0 (Negative)	LVI = 1 (Positive)	*P*
Age	122	49.99(10.17)	48.93(9.34)	0.566
History of giving birth	122	80	42	0.488
No	5	4(80.00)	1(20.00)	
Yes	117	76(64.96)	41(35.04)	
History of abortion	122	80	42	0.560
No	86	55(63.95)	31(36.05)	
Yes	36	25(69.44)	11(30.56)	
nipple discharge	122	80	42	
No	120	80(66.67)	40(33.33)	
Yes	2	0(0.00)	2(100.00)	
CA153^a^	121	79	42	0.514
Negative	117	77(65.81)	40(34.19)	
Positive	4	2(50.00)	2(50.00)	
History of related illness	122	80	42	0.488
No	117	76(64.96)	41(35.04)	
Yes	5	4(80.00)	1(20.00)	
ER	122	80	42	0.062
Negative	39	21(53.85)	18(46.15)	
Positive	83	59(71.08)	24(28.92)	
PR	122	80	42	0.0591
Negative	47	26(55.32)	21(44.68)	
Positive	75	54(72.00)	21(28.00)	
HER-2	122	80	42	0.994
Negative	32	21(65.63)	11(34.38)	
Positive	90	59(65.56)	31(34.44)	
E-cad^a^	87	56	31	0.100
Negative	6	2(33.33)	4(66.67)	
Positive	81	54(66.67)	27(33.33)	
Ki-67	122	80	42	**0.012**
Low	27	20(74.07)	7(25.93)	
Moderate	36	29(80.56)	7(19.44)	
High	59	31(52.54)	28(47.46)	
P53	122	80	42	0.126
Negative	40	30(75.00)	10(25.00)	
Positive	82	50(60.98)	32(39.02)	
Amount of fibroglandular tissue	122	80	42	0.431
Almost entirely fat/Scattered fibroglandular tissue	49	33(67.35)	16(32.65)	
Heterogeneous fibroglandular tissue	54	37(68.52)	17(31.48)	
Extreme fibroglandular tissue	19	10(52.63)	9(47.37)	
Size	122	80	42	0.094
≤2cm	79	56(70.89)	23(29.11)	
>2cm	43	24(55.81)	19(44.19)	
Single/multiple	122	80	42	0.746
Single	95	63(66.32)	32(33.68)	
Multiple	27	17(62.96)	10(37.04)	
Lesions	122	80	42	0.8855
Mass	65	43(66.15)	22(33.85)	
Mass/calcification	57	37(64.91)	20(35.09)	
Location	122	80	42	0.879
Outer upper	73	49(67.12)	24(32.88)	
Outer lower	5	4(80.00)	1(20.00)	
Lower inner	15	10(66.67)	5(33.33)	
Upper inner	15	9(60.00)	6(40.00)	
Central area	14	8(57.14)	6(42.86)	
Mass shape	122	80	42	0.160
Lobulated	16	8(50.00)	8(50.00)	
Irregular	106	72(67.92)	34(32.08)	
Mass Margin	122	80	42	0.088
Smooth	6	2(33.33)	4(66.67)	
Rough	116	78(67.24)	38(32.76)	
Boundary	122	80	42	0.714
Clear	69	47(68.12)	22(31.88)	
Obscure	27	16(59.26)	11(40.74)	
Shield	26	17(65.38)	9(34.62)	
Calcification^a^	56	36	20	0.279
Vague and amorphous	25	18(72.00)	7(28.00)	
Fine polymorphous	31	18(58.06)	13(41.94)	
Interstitial edema	122	80	42	**0.013**
No	110	76(69.09)	34(30.91)	
Yes	12	4(33.33)	8(66.67)	
Subcutaneous fat	122	80	42	0.697
Clear	101	67(66.34)	34(33.66)	
Muddy	21	13(61.90)	8(38.10)	
Thicken Skin	122	80	42	**< 0.001**
No	86	65(75.58)	21(24.42)	
Yes	36	15(41.67)	21(58.33)	
Nipple retraction	122	80	42	0.073
No	98	68(69.39)	30(30.61)	
Yes	24	12(50.00)	12(50.00)	
Axillary lymph node enlargement	122	80	42	0.166
No	77	54(70.13)	23(29.87)	
Yes	45	26(57.78)	19(42.22)	

^a^Missing value exists

*LVI* Lymphovascular invasion

### Digital mammography findings

**Fig. 1:**
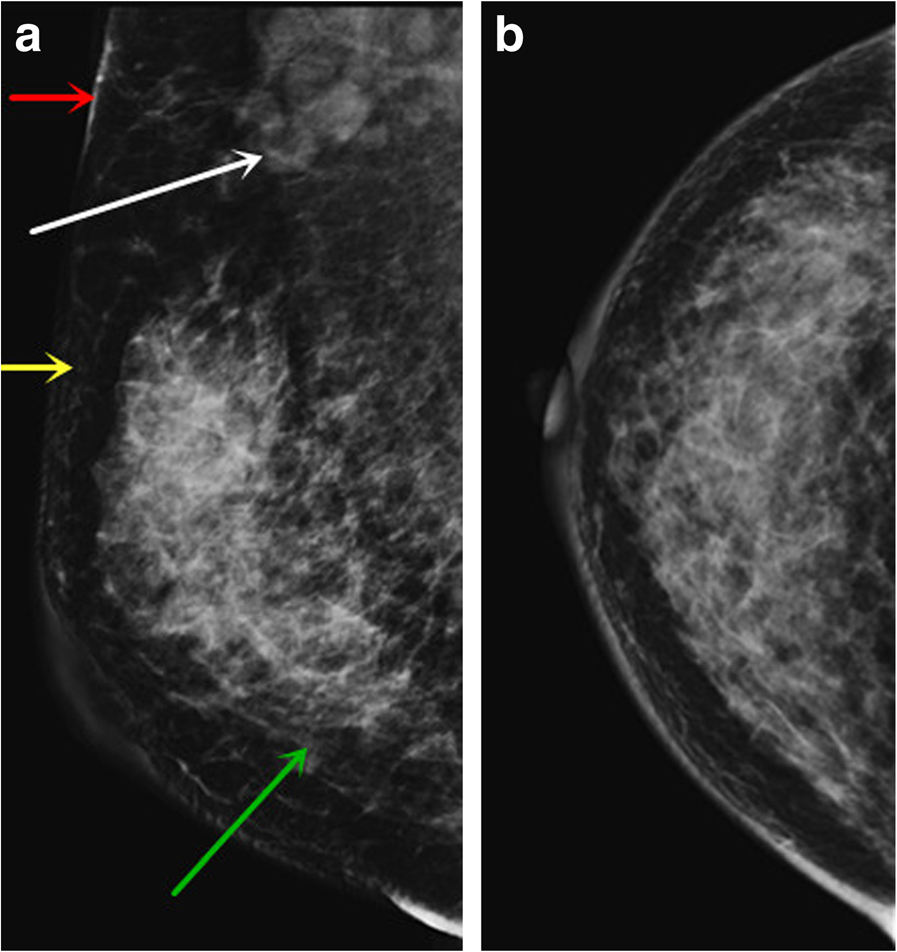
Female, LVI positive. The MLO position (**a**) and CC position (**b**) of digital mammography showed an irregular upper mass with blurred boundaries in the upper outer quadrant of the right breast. Interstitial edema (green arrow), blurring of subcutaneous fat layer (yellow arrow), skin thickening (red arrow), and axillary adenopathy (white arrow) were observed in the right breast

**Fig. 2:**
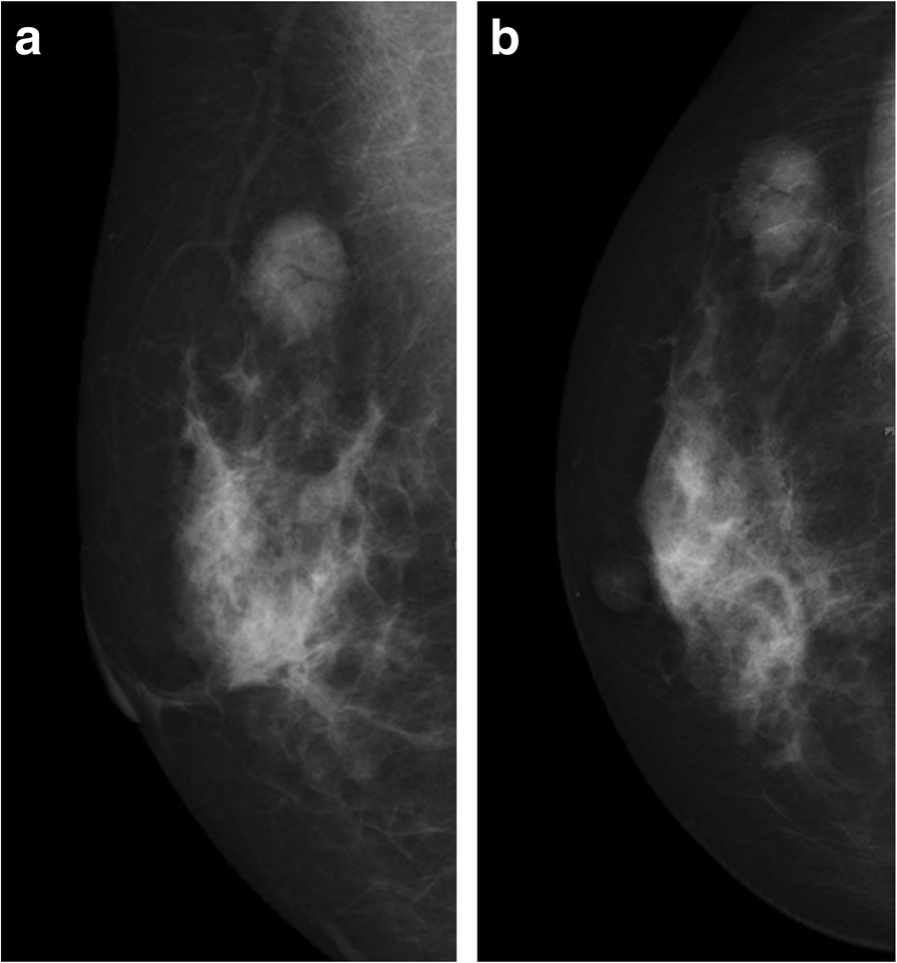
Female, LVI negative. The MLO position (**a**) and CC position (**b**) of the digital mammography showed a circular mass with smooth edges and clear boundaries in the upper outer quadrant of the right breast. No accompanying patterns (such as interstitial edema or skin thickening) were seen

Details are shown in Table 1. Differences in interstitial edema (*P* = 0.013, Figs. 1 and 2) and skin thickening (*P* = 0.000, Figs. 1 and 2) between the two groups were statistically significant. No statistically significant intergroup differences were seen in other imaging features, such as fibroglandular tissue density (*P* = 0.431), radiological diameter (*P* = 0.094), mass number (*P* = 0.746), lesion number (*P* = 0.8855), location (*P* = 0.879), mass shape (*P* = 0.160), mass margin (*P* = 0.088), boundary (*P* = 0.714), calcification (*P* = 0.279), subcutaneous fat (*P* = 0.697), nipple retraction (*P* = 0.073) and axillary adenopathy (*P* = 0.166).

Risk factor analysis results are shown in Table 2. Multiple factor analysis showed that there are three independent risk factors for LVI: interstitial edema (odds ratio [OR] = 12.610; 95% confidence interval [CI]: 1.061–149.922; *P* = 0.045), subcutaneous fat (OR = 0.081; 95% CI: 0.012–0.645; *P* = 0.017) and skin thickening (OR = 9.041; 95% CI: 2.553–32.022; *P* = 0.001).

**Table 2:** Univariate and multivariate analysis

Factors	Univariate analysis		Multivariate analysis	
	OR (95% CI)	*P*	OR (95% CI)	*P*
Interstitial edema	4.471(1.260,15.864)	**0.020**	**12.610(1.061,149.922)**	**0.045**
Subcutaneous fat	1.213(0.458,3.207)	0.698	**0.081(0.012,0.645)**	**0.017**
Thicken Skin	4.333(1.899,9.891)	**0.000**	**9.041(2.553,32.022)**	**0.001**
ER	0.475(0.216,1.044)	0.064	1.595(0.252,10.084)	0.62
PR	0.481(0.224,1.034)	0.061	0.508(0.085,3.035)	0.458
HER-2	1.003(0.429,2.345)	0.994	1.436(0.462,4.466)	0.532
Ki-67				
Moderate	0.690(0.209,2.273)	0.541	0.382(0.094,1.551)	0.178
High	2.581(0.948,7.022)	0.063	1.298(0.350,4.809)	0.696
P53	1.920(0.827,4.457)	0.129	2.115(0.739,6.050)	0.163
Amount of fibroglandular tissue				
Heterogeneous fibroglandular tissue	0.948(0.414,2.179)	0.899	1.439(0.491,4.215)	0.507
Extreme fibroglandular tissue	1.856(0.630,5.469)	0.262	3.773(0.707,20.154)	0.12
Size	1.928(0.890,4176)	0.096	0.921(0.294,2.891)	0.889
Nipple retraction	2.267(0.914,5.621)	0.077	1.299(0.281,5.991)	0.738
Single/multiple	1.158(0.476,2.819)	0.746	1.183(0.336,4.165)	0.793
Lesions	1.057(0.500,2.233)	0.885	0.712(0.256,1.979)	0.514
Mass shape	0.472(0.163,1.365)	0.166	0.624(0.128,3.047)	0.56
Margin	0.224(0.043,1.389)	0.112	0.424(0.032,5.630)	0.516
Boundary				
Obscure	1.469(0.586,3.684)	0.413	1.483(0.439,5.007)	0.526
Shield	1.131(0.436,2.935)	0.800	0.997(0.246,4.043)	0.997
Axillary lymph node enlargement	1.716(0.797,3.694)	0.168	0.909(0.295,2.805)	0.868
E-cad	0.250(0.043,1.452)	0.122		
Location				
Outer lower	0.510(0.054,4.819)	0.557		
Lower inner	1.021(0.314,3.320)	0.973		
Upper inner	1.361(0.434,4.267)	0.597		
Central area	1.531(0.477,4.913)	0.474		
Calcification	1.857(0.601,5.734)	0.166		
Family history	1.927(0.117,31.602)	0.646		
History of giving birth	2.158(0.233,19.947)	0.498		
History of abortion	0.781(0.339,1.799)	0.561		
CA15–3	1.925(0.261,14.179)	0.52		
History of related illness	0.463(0.050,4.284)	0.498		

Table 3 shows the sensitivity, specificity, accuracy, positive predictive value and negative predictive value of the three independent risk factors in LVI prediction. The specificity of LVI prediction was as high as 98.8% when they were applied together.

**Table 3:** Diagnostic performance

Methods	Sensitivity	Specificity	Accuracy	PPV	NPV
Interstitial edema	19.0(8/42)	95.0(76/80)	68.9(84/122)	66.7(8/12)	69.1(76/110)
Thicken Skin	50.0(21/42)	81.3(65/80)	70.5(86/122)	58.3(21/36)	75.6(65/86)
Subcutaneous fat	19.0(8/42)	83.8(67/80)	61.2(75/122)	38.1(8/21)	66.3(67/101)
All factors	14.3(6/42)	98.8(79/80)	69.7(85/122)	85.7(6/7)	68.7(79/115)

## Discussion

LVI or intralymphovascular tumor emboli is closely related to the adverse outcome of many malignant tumors [15–17]. As a risk factor for recurrent breast cancer following modified radical mastectomy, lymphovascular tumor emboli, especially lymphatic tumor emboli, has been included in the St Gallen consensus for breast cancer [18]. Karlsson et al. [19] reported that the failure rate of chemotherapy was higher in breast cancer patients with LVI than those without. Shen et al. [20] found that lymphovascular tumor emboli promoted recurrence and distant metastasis of local tumors. Therefore, presence of lymphovascular tumor emboli is a reliable indicator for distant metastasis of breast cancer and an important factor influencing overall survival. This article aimed to predict the risk of LVI of breast cancer using various digital mammography features.

There are no statistically significant differences in age, childbearing history, miscarriage history, family history and other medical history between the LVI positive group and the LVI negative group. Nulliparity, miscarriage, and family history of breast cancer are not associated with increase of LVI occurrence.

CA153 is a tumor marker first discovered in breast cancer cells. Its specificity is relatively high in diagnosis of breast cancer. Diagnostic sensitivity of CA153 increases from 66 to 80% as breast cancer advances [21]. However, in this study, there are only 4 cases of positive CA153 (3.3%). Though there are no statistical significant differences in ER, PR, Her-2, E-cad, and P53 between the two groups, the LVI positive group have more cases of high Ki-67 expression level (> 30%) than the LVI negative group and the difference is statistically significant (*P* = 0.012). Some literature have confirmed that Ki-67 is associated with tumor differentiation, LVI, metastasis, and recurrence [22–24].

There are no statistically significant differences between the two groups in mammographic density, location, number, radiological diameter, shape, margin, boundary, calcification. Therefore, the above factors cannot be used to predict LVI. However, intergroup differences in interstitial edema and skin thickening are statistically significant (*P* = 0.013 and 0.000, respectively). In addition, multivariate analysis demonstrates that interstitial edema, blurring of subcutaneous fat, and skin thickening are independent risk factors for LVI (*P* = 0.045, 0.017 and 0.001, respectively). In clinical work-up, physicians should be highly alerted about LVI occurrence when digital mammography reveals the above three phenomena. Even if lymph node metastasis is negative in sentinel lymph node and axillary lymph node biopsies, there is possibility that breast cancer cells has infiltrated surrounding vessels but not metastasized upwards to the axillary lymph nodes yet. Besides, modification of postoperative adjuvant therapy should be considered to reduce risk of recurrence and distant metastasis, and thus to prolong survival.

We believed that the axillary lymph nodes shown on digital mammography could be used to predict LVI. However, there are no differences between the two groups in axillary lymph nodes (*P* = 0.166). One possible explanation might be that digital mammography failed to reveal axillary lymph nodes. In addition, an enlarged (> 1 cm) or complete lymph node does not always suggest metastasis. There is possibility of reactive hyperplasia.

## Conclusions

There is statistically significant difference in Ki-67 between the LVI positive group and the LVI negative group. Interstitial edema, blurring of subcutaneous fat and skin thickening are independent risk factors for LVI (*P* = 0.045, 0.017, and 0.001, respectively). When the three imaging features are applied together, the specificity of LVI prediction is as high as 98.8%.

## Data Availability

Data to replicate findings are in the Figures and Tables of the main paper. Due to patient privacy protection, any additional materials of the study are only available upon individual request directed to the corresponding author.

## Abbreviations

LVI: Lymphovascular invasion
DM: Digital mammography
RCC: Right craniocaudal
LCC: Left craniocaudal
RMLO: Right mediolateral oblique
LMLO: Left mediolateral oblique
